# Research priorities for nutrition of school-aged children and adolescents in low- and middle-income countries

**DOI:** 10.1371/journal.pone.0280510

**Published:** 2023-01-20

**Authors:** Natasha Lelijveld, Stephanie V. Wrottesley, Dina Aburmishan, Elena C. Hemler, Netsanet Fentahun, Zakari Ali, Rachael Menezes, Marion Roche, Deepika Sharma, Kerri Wazny, David A. Ross, Vani Sethi, Emily Mates

**Affiliations:** 1 Emergency Nutrition Network, Kidlington, United Kingdom; 2 Department of Population Health, London School of Hygiene and Tropical Medicine, London, United Kingdom; 3 Bureau for Humanitarian Assistance (BHA), USAID, Washington, D.C., United States of America; 4 Department of Global Health & Population, Harvard T.H. Chan School of Public Health, Boston, MA, United States of America; 5 Department of Nutrition and Dietetics, School of Public Health, College of Medicine and Health Sciences, Bahir Dar University, Bahir Dar, Ethiopia; 6 MRC Unit The Gambia, London School of Hygiene and Tropical Medicine, Banjul, The Gambia; 7 Nutrition International, Ottawa, Canada; 8 UNICEF Head Quarters, New York, NY, United States of America; 9 Global Disease Epidemiology and Control, Johns Hopkins Bloomberg School of Public Health, Baltimore, MD, United States of America; 10 Department of Epidemiology and Public Health, Stellenbosch University, Stellenbosch, South Africa; 11 UNICEF India, New Delhi, India; University of Ibadan, NIGERIA

## Abstract

**Purpose:**

A lack of data, intervention studies, policies, and targets for nutrition in school-age children (SAC) and adolescents (5-19 years) is hampering progress towards tackling malnutrition. To stimulate and guide further research, this study generated a list of research priorities.

**Methods:**

Using the Child Health and Nutrition Research Initiative (CHNRI) method, a list of 48 research questions was compiled and questions were scored against defined criteria using a stakeholder survey. Questions covered all forms of malnutrition, including micronutrient deficiencies, thinness, stunting, overweight/obesity, and suboptimal dietary quality. The context was defined as research focused on SAC and adolescents, 5 to 19 years old, in low-and middle-income countries, that could achieve measurable results in reducing the prevalence of malnutrition in the next 10 years.

**Results:**

Between 85 and 101 stakeholders responded per question. Respondents covered a broad geographical distribution across 38 countries, with the largest proportion focusing on work in East and Southern Africa. Of the research questions ranked in the top ten, half focused on delivery strategies for reaching adolescents and half on improving existing interventions. There were few differences in the ranked order of questions between age groups but those related to in-school children and adolescents had higher expert agreement than those for out-of-school adolescents. The top ranked research question focused on tailoring antenatal and postnatal care for pregnant adolescent girls.

**Conclusion:**

Nutrition programmes should incorporate implementation research to inform delivery of effective interventions to this age group, starting in schools. Academic research on the development and tailoring of existing nutrition interventions is also needed; specifically, on how to package multisectoral programmes and how to better reach vulnerable and underserved sub- groups, including those out of school.

## Introduction

Nutritional status during middle childhood (5-9 years) and adolescence (10-19 years) affects physical, cognitive and social development, with implications throughout the life course as well as for future generations [1]. Growth and development across these periods establish adult health trajectories via biological and behavioural pathways. Approximately 20% of adult height, 60% of skeletal mass, and half of adult body weight is achieved between 10 and 19 years of age [2]. To support this rapid growth and development, children and adolescents have increased needs for energy, protein, iron, and other micronutrients [3].

An estimated 21 million girls aged 15–19 years in low- and middle-income countries (LMICs) become pregnant every year [4]. This disrupts their linear growth and results in even greater dietary requirements to support their own health and the health of their child [5]. Stunted children 5-9 years often enrol late in school, and on-going malnutrition affects their ability to learn and concentrate [6]. Overweight during middle childhood and adolescence increases the risk of developing non-communicable diseases in adulthood, which have major impacts on quality of life and survival, as well as economic costs [7]. Despite its importance, nutrition during middle childhood and adolescence (5-19 years) has been relatively neglected, with much of the global focus being on children under five years of age, or “the first 1000 days” of life.

In LMICs, school-aged children (SAC) and adolescents face a range of nutrition challenges, including thinness, stunting, overweight and obesity, anaemia, and other micronutrient deficiencies [8, 9]. However, prevalence data for this age group are sparse. There is an especially large data gap for middle childhood (5-9 years) and early adolescence (10-14 years). Where there is data, it is often not disaggregated into age sub-categories, making it difficult to understand nuances across this large and heterogenous age group. For example, girls 15-19 years are often only included as part of the broader ‘women of reproductive age’ (15-49 years) group. Major policy gaps, and a lack of both intervention studies and international and national targets for nutrition in SAC and adolescents further hampers progress [10, 11].

Given these large and diverse gaps in knowledge, programming, and policy, more evidence is needed to inform programming and policy; this also requires greater investment by donors into research on this topic. To help progress this agenda, we undertook a research prioritisation exercise for tackling malnutrition in SAC and adolescents in LMICs, with the objective of stimulating and guiding future research to inform policies and programming.

## Materials and methods

### Survey development

This research prioritisation (RP) exercise followed the Child Health and Nutrition Research Initiative (CHNRI) method developed to assist stakeholders in prioritising health research investments and described in detail elsewhere [12]. The method involves identifying and listing many possible research questions within a well-defined context. The questions are then scored according to a systematic and transparent “4D” framework. The 4Ds are defined as follows and, in our case, adapted specifically to nutrition:

- ***Description*:** research to assess the burden of the problem, its determinants, and effectiveness of interventions to address the problem
- ***Delivery*:** research to improve how nutrition interventions are delivered, financed, and taken-up
- ***Development*:** research to improve nutrition interventions that already exist
- ***Discovery*:** research that leads to innovation i.e., entirely new nutrition interventions

The context and scope of this RP exercise were as follows:

- **Population**: SAC and adolescents, 5-19 years old, disaggregated by age group (middle childhood (5-9 years), early adolescence (10-14 years), late adolescence (15-19 years)), in school vs. out of school, and separate questions for pregnant adolescent girls.
- **Disease**: all forms of malnutrition, including micronutrient deficiencies (vitamin A, vitamin D, iron, all cause anaemia, zinc, iodine, and calcium), thinness (low body mass index (BMI)-for-age), stunting (low height-for-age), overweight/obesity (high BMI-for-age), and suboptimal dietary quality.
- **Geography**: LMICs, including research at sub-national, national, regional, or global level.
- **Timescale**: to achieve measurable results in reducing the prevalence of malnutrition in the next 10 years

An initial list of 71 research questions was compiled, informed by key literature (reviews, metanalyses, Lancet series, and opinion pieces only) published in the past seven years (2014-2021) (39 articles) and several other resources: a systematic literature review of primary research conducted for each of the UNICEF regions in 2020 (991 articles) [13]; and an open stakeholder survey with the option to submit suggested research gaps (133 respondents) [14]. An expert group of 21 leading specialists in adolescent nutrition, with diverse representation, collectively refined and reduced the list to 48 questions (39 general and nine specific to pregnant adolescents) which made up the final survey. The group also selected four priority-setting criteria from those recommended by the CHNRI process, against which the questions should be scored (**Table 1**).

**Table 1:** Criteria used to prioritise research questions.

**Answerability**	This research question is *answerable*
**Effectiveness**	This research could result in an intervention that is *effective* for preventing or managing malnutrition in school-aged children (SAC) and adolescents * *
**Deliverability**	This research could result in an intervention that is *deliverable*
**Equity**	This research could result in an intervention that *improves equity* amongst SAC and adolescents

### Survey dissemination

The survey was made available in English, French, and Spanish and was published online from 20^th^ July to 18^th^ September 2021. The order of question sections was randomised to ensure a similar response rate per section. The survey link was circulated via the expert group, the Emergency Nutrition Network (ENN) Global Adolescent Nutrition Network (GANN) and email list from a recent webinar, en-net forum, UNICEF country offices, World Food Programme (WFP) country offices, and ENN social media platforms.

### Survey completion and analysis

Survey respondents were first asked basic demographic information including their work organisation, the geographical focus of their work, and their age category. For each research question, respondents were required to assess whether each of the criteria were met by indicating “Yes” (allocated 1 point), “Undecided” (0.5 points), “No” (0 points), or “Insufficiently informed” (no input). An individual research priority score (RPS) of 0–100% was calculated for each criterion per research question; from this, an overall RPS for each question was computed (the mean of the four RPSs for each criterion) and used to rank the questions. The level of agreement between respondents’ answers was assessed using average expert agreement (AEA) as follows:

$$AEA=\frac{1}{4}X\;\sum_{q=1}^{4}\left(\frac{N(number\;of\;scorers\;with\;most\;frequent\;answer)}{N(number\;of\;scorers\;who\;provided\;any\;answer)}\right)$$

where q is a question that experts are being asked to evaluate and 4 is the number of possible answers.

Respondents were also asked to rank some questions by age group (5-9 years, 10-14 years, 15-19 years), by in-school vs. out-of-school, and pregnant/non-pregnant adolescents, so that results could be disaggregated by these categories.

### Ethical approval

As is standard for CHNRI exercises [12], this project did not require formal ethical committee review since the work does not involve medical research on human subjects, no personal or sensitive data was used and respondents were professionals rather than patients. All respondents acknowledged their informed, voluntary participation in the exercise at the start of the survey and no special informed consent was required. All responses were anonymised, and respondents were informed that data would be used for publication.

## Results

### Characteristics of respondents

**Fig 1:**
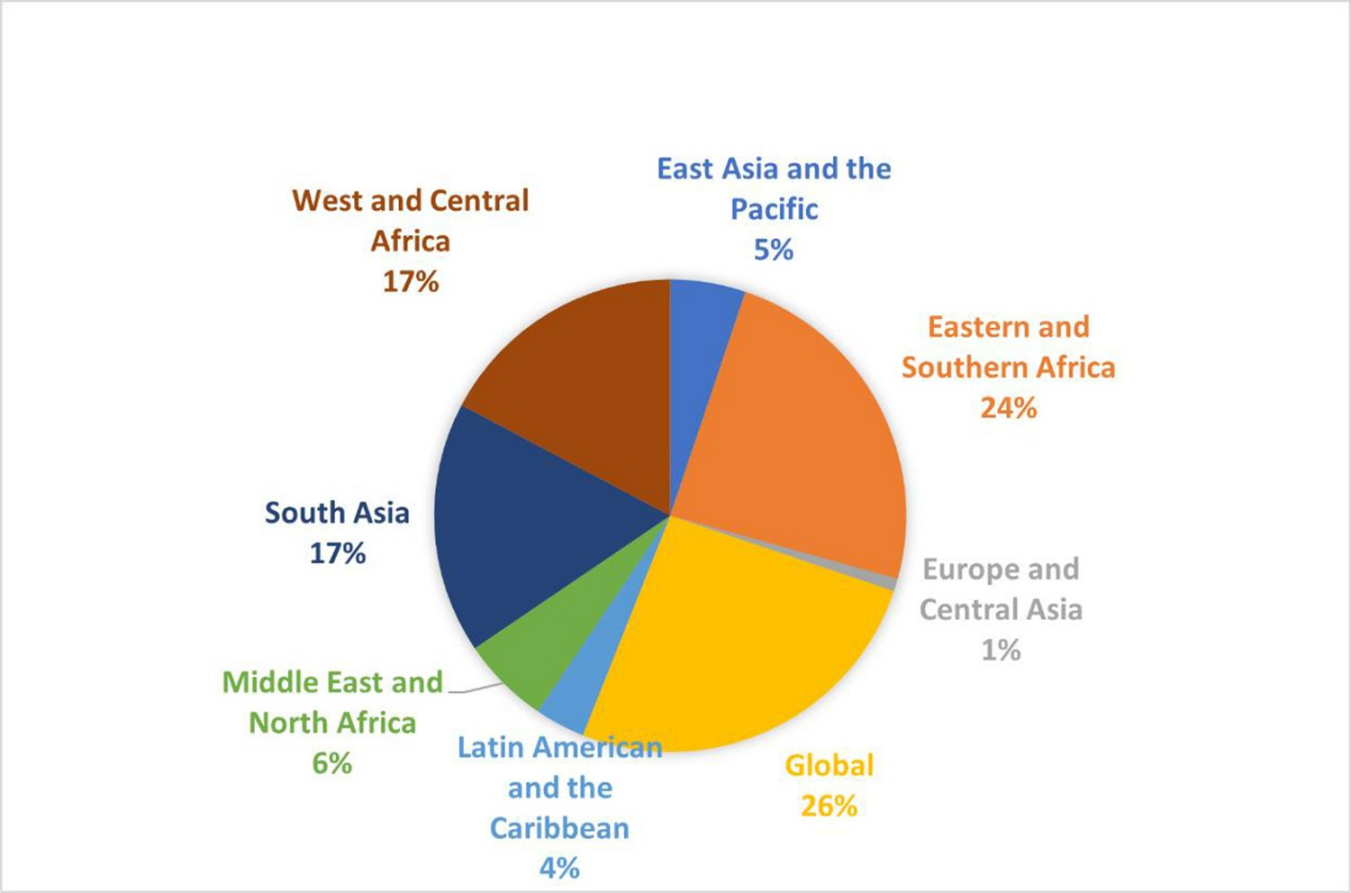
Regional representation of respondents. Regions identified by respondents as the main focus of their work; if respondents work across multiple regions, they could select ‘global’.

**Fig 2:**
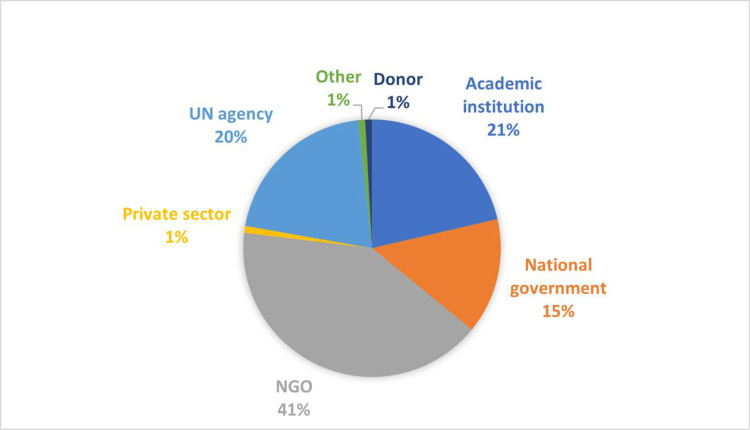
Type of organisations represented by respondents. NGO = non-government organisation.

A total of 285 people registered for the survey; 116 respondents completed at least one section of the survey (six in French, three in Spanish). Question order was randomised so that incomplete surveys could be included; the lowest number of responses per question was 85 and the largest was 101. Approximately 3% (4/116) of respondents were younger than 25. Respondents reported that their work focused on 38 unique countries and represented a broad range of organisations, with the majority being programme implementors working for non-government organisations (NGOs) (41%) and UN organisations (20%), and then researchers from academic institutions (21%) (**Figs 1 and 2**). Countries in the Europe and Central Asia region, East Asia and Pacific region, and Latin America and the Caribbean region were the least represented.

### Overall top ten ranked research questions

Half (5/10) of the research questions ranked in the top ten focused on delivery strategies for reaching adolescents and the other half (5/10) on improvement of existing interventions (**Table 2**). None of the questions ranked in the top ten focused on describing the problem and/or solutions, or discovery of innovative solutions. However, it is useful to note that scores were generally high, with a small range across most research questions (see full list in **S1 Table**). The delivery-related research questions in the top ten focused on: delivering effective interventions in schools; defining other effective delivery platforms, including those for reaching high-risk subgroups; effectively involving SAC and adolescents in defining their own context-specific solutions; and creating effective behaviour change communication strategies. The development-related research questions focused on: how to adapt antenatal and postnatal care to effectively support pregnant adolescents; how to improve local food systems to support access to healthy diets in schools; how to better tailor interventions for girls and boys; how to evaluate the impact of peer education programmes; and what combination of existing interventions is effective at addressing malnutrition. The five top ranked questions overall had high average expert agreement scores (>80%).

**Table 2:** Overall top ten ranked research questions across all sub-categories.

RANK	RESEARCH QUESTION	RESEARCH AREA	A	E	D	EQ	RPS	AEA
**1**	How should antenatal and postnatal care interventions be adapted to effectively and cost-effectively support the specific health and nutritional needs of pregnant adolescents?	Development: pregnancy	90.3	93.8	93.2	93.7	92.8	85.9
**2**	What strategies are effective for delivering interventions in schools to improve quality of diets and nutritional outcomes of SAC and adolescents?	Delivery: How best to reach SAC and adolescents?	94.8	95.4	96.0	80.6	91.7	86.3
**3**	What strategies are effective at involving SAC and adolescents in defining their own context-specific solutions to nutrition problems, and does their involvement result in more effective interventions?	Delivery: How best to reach SAC and adolescents?	94.3	93.1	88.7	89.2	91.3	85.0
**4**	What are effective, context-specific, behaviour change communication strategies to improve diets and nutritional status of SAC and adolescents?	Delivery: How best to reach SAC and adolescents?	89.8	87.5	94.3	84.6	89.0	82.4
**5**	What improvements can be made to local food systems to support access to healthy diets in schools?	Development - existing nutrition sensitive interventions	91.4	92.5	87.5	84.0	88.8	81.0
**6**	Does sex and/or gender impact the response to nutrition interventions (e.g., obesity prevention interventions), and how can interventions be better tailored to girls and boys?	Development - existing nutrition sensitive interventions	91.7	90.1	85.9	87.6	88.8	79.5
**7**	What are the optimal delivery platforms (health, education, social protection, media/technology etc.) for effective uptake of nutrition interventions for SAC and adolescents, taking into account scale, sustainability and youth engagement?	Delivery: How best to reach SAC and adolescents with interventions, including sub-groups?	90.4	90.4	89.7	84.3	88.7	79.2
**8**	What are the optimal delivery platforms for reaching the sub-groups of SAC and adolescents identified as highest priority?	Delivery: How best to reach SAC and adolescents?	89.3	88.8	87.6	86.5	88.1	79.3
**9**	What is the impact of peer education programmes on nutrition of adolescents and SAC in different contexts?	Development – existing nutrition sensitive interventions	91.7	87.9	88.9	80.4	87.2	77.3
**10**	What combined package of existing interventions is effective at addressing malnutrition in SAC and adolescents?	Development – packages of existing interventions	85.7	92.2	88.9	81.2	87.0	75.8

A=answerability, E=effectiveness, D=deliverability, Eq=equity, RPS=research priority score, AEA= average expert agreement. Ranking based on RPS.

### Top ranked questions disaggregated by age category

For age-disaggregated questions, the top ten ranked questions were similar across the three age categories (**Table 3**). The top five ranked questions remained the same and the next five only differed slightly in the order of priority. The main differences related to the question on drivers of food choice, which was ranked as a higher priority for the oldest age category than the younger categories and the question on optimal school meals, which was ranked comparatively higher for the youngest age category.

**Table 3:** Top ten ranked research questions disaggregated by age category.

RANK	AGE RANGE	RESEARCH QUESTION	RESEARCH AREA	A	E	D	EQ	RPS	AEA
**1**	5-9	What improvements can be made to local food systems to support access to healthy diets in schools?	Development – existing nutrition sensitive interventions	91.4	92.5	87.5	84.0	88.8	81.0
	10-14			91.4	92.5	87.5	84.0	88.8	81.0
	15-19			91.4	92.5	87.5	84.0	88.8	81.0
**2**	5-9	Does sex and/or gender impact the response to nutrition interventions (e.g., obesity prevention interventions), and how can interventions be better tailored to girls and boys?	Development – existing nutrition sensitive interventions	91.7	90.1	85.9	87.6	88.8	79.5
	10-14			91.7	90.1	85.9	87.6	88.8	79.5
	15-19			91.7	90.1	85.9	87.6	88.8	79.5
**3**	5-9	What is the impact of peer education programmes on nutrition of adolescents and SAC in different contexts?	Development – existing nutrition sensitive interventions	91.7	87.8	88.9	80.4	87.2	77.3
	10-14			91.7	87.8	88.9	80.4	87.2	77.3
	15-19			91.7	87.8	88.9	80.4	87.2	77.3
**4**	5-9	What combined package of existing interventions is effective at addressing malnutrition in SAC and adolescents?	Development – packages of existing interventions	85.7	92.2	88.9	81.2	87.0	75.8
	10-14			85.7	92.2	88.9	81.2	87.0	75.8
	15-19			85.7	92.2	88.9	81.2	87.0	75.8
**5**	5-9	What is the feasibility, effectiveness and cost-effectiveness of interventions in the school food-environment on multiple forms of malnutrition, such as applying limitations on marketing around schools?	Development – existing nutrition sensitive interventions	85.7	89.3	88.4	81.1	86.1	75.3
	10-14			85.7	89.3	88.4	81.1	86.1	75.3
	15-19			85.7	89.3	88.4	81.1	86.1	75.3
**6**	5-9	What standard indicators should be used to assess impact of nutrition interventions on school achievement, productivity, and wellbeing (e.g., mental, social, spiritual health) in SAC and adolescents?	Discovery - advances in assessment	88.5	87.6	85.0	81.5	85.6	74.1
**6**	10-14			88.5	87.6	85.0	81.5	85.6	74.0
**7**	15-19			88.5	87.6	85.0	81.5	85.6	74.1
**15**	5-9	What are the determinants and drivers of food choices among SAC and adolescents (e.g., influences of family members, marketing practices), by context and sub-groups?	Description - determinants of diets	83.2	79.3	77.2	79.3	82.1	70.7
**10**	10-14			88.6	85.1	81.8	82.4	84.5	73.3
**6**	15-19			90.3	86.9	81.3	85.2	85.9	76.4
**7**	5-9	What is the effectiveness of coupling food production and the promotion of nutritious food interventions at school/household/ community level on nutrition outcomes in SAC and adolescents?	Development – packages of existing interventions	86.7	88.7	82.9	81.6	85.0	74.4
**7**	10-14			86.7	88.7	82.9	81.6	85.0	74.4
**8**	15-19			86.7	88.8	82.9	81.6	85.0	74.4
**8**	5-9	What is the optimal nutritional quality, quantity, and timing of school-meals for improving nutritional outcomes cost-effectively, in different contexts?	Development – existing nutrition specific interventions	85.2	87.9	84.7	79.1	84.2	73.3
**11**	10-14			85.2	87.9	84.7	79.1	84.2	73.3
**9**	15-19			85.2	87.9	84.7	79.1	84.2	73.3
**9**	5-9	What are the determinants of undernutrition, overweight/obesity and micronutrient deficiencies in SAC and adolescents, disaggregated by sex, and context?	Description - determinants of poor nutritional status	84.1	85.9	80.9	85.5	84.1	73.6
**8**	10-14			83.8	87.5	81.5	86.5	84.8	72.6
**10**	15-19			80.9	88.1	80.7	86.4	84.0	71.9
**10**	5-9	What risk factors impact the nutritional status of SAC and adolescents, including those in humanitarian contexts?	Description - determinants of poor nutritional status	86.0	84.4	80.9	84.2	83.9	71.3
**9**	10-14			86.2	86.4	81.3	84.0	84.5	70.6
**12**	15-19			85.9	84.3	78.9	84.1	83.3	69.8

*while these questions were disaggregated by age group, the scores across the 3 age categories were the same A=answerability, E=effectiveness, D=deliverability, Eq=equity. RPS=research priority score, AEA= average expert agreement. Ranking based on RPS.

### In-school and out-of-school SAC and adolescents

The delivery questions were disaggregated for in-school and out-of-school children and adolescents. The top ranked question for those in school related to effective delivery of school-based interventions, whereas the top ranked question for those out-of-school focused on optimal alternative delivery platforms (health, education, social protection, media/technology, etc.) for effective uptake of nutrition interventions (**Table 4**). The other questions were similarly ranked for those in- and out-of-school. Of all the top ranked questions, expert agreement was lowest for those questions pertaining to out-of-school adolescents.

**Table 4:** Top five ranked research questions disaggregated for those in- and out-of-school.

SCHOOL STATUS	RANK	RESEARCH QUESTION	RESEARCH AREA	A	E	D	EQ	RPS	AEA
In sch	1	What strategies are effective for delivering interventions in schools to improve quality of diets and nutritional outcomes of SAC and adolescents?	Delivery: How best to reach SAC and adolescents?	94.8	95.4	96.0	80.6	91.7	86.3
Out of sch	9			65.7	64.8	63.1	61.5	63.8	55.9
In sch	2	What strategies are effective at involving SAC and adolescents in defining their own context-specific solutions to nutrition problems, and does their involvement result in more effective interventions?	Delivery: How best to reach SAC and adolescents?	94.3	93.1	88.7	89.2	91.3	85.0
				84.4	85.8	79.8	85.8	84.0	71.0
Out of sch	2								
In sch	3	What are effective, context-specific, behaviour change communication strategies to improve diets and nutritional status of SAC and adolescents?	Delivery: How best to reach SAC and adolescents?	89.8	87.5	94.3	84.6	89.0	82.4
Out of sch	3			84.8	82.0	86.1	82.3	83.8	71.6
In sch	4	What are the optimal delivery platforms (health, education, social protection, media/technology etc.) for effective uptake of nutrition interventions for SAC and adolescents, taking into account scale, sustainability and youth engagement?	Delivery: How best to reach SAC and adolescents?	90.4	90.4	89.7	84.3	88.7	79.2
Out of sch	1			83.3	86.1	84.6	82.9	84.2	70.7
In sch	5	What are the optimal delivery platforms for reaching the sub-groups of SAC and adolescents identified as highest priority?	Delivery: How best to reach SAC and adolescents?	89.3	88.8	87.6	86.5	88.1	79.2
Out of sch	4			78.8	80.5	78.3	82.2	80.0	66.6
In sch	6	What is the most acceptable and effective strategy for implementing the WHO guideline of weekly iron-folic acid supplementation, including adherence, optimal dose and duration, depending on baseline anaemia prevalence?	Delivery: How best to reach SAC and adolescents?	85.5	85.4	86.9	81.5	84.8	75.0
Out of sch	5			75.2	79.2	79.1	80.0	78.4	64.0

Sch=school. A=answerability, E=effectiveness, D=deliverability, Eq=equity. RPS=research priority score, AEA= average expert agreement. Ranking based on RPS.

### Pregnant adolescents

Nine questions in the survey specifically focused on pregnant adolescents. The top five according to RPS are presented in **Table 5**. Of the top five, two were concerned with description of the problem, two with improvement of existing interventions, and one with delivery of interventions. The description questions focused on describing the risk factors of malnutrition during pregnancy, including quantifying the benefit(s) of delaying pregnancy. The development questions focused on tailoring existing antenatal and postnatal care interventions to be more effective and cost-effective for pregnant adolescents. The delivery question focused on the most acceptable and effective strategy for implementing daily/weekly multiple micronutrient supplementation for pregnant adolescents.

**Table 5:** Top five ranked research questions for pregnant adolescents specifically.

RANK	RESEARCH AREA	RESEARCH QUESTION	A	E	D	EQ	RPS	AEA
**1**	Development	How should antenatal and postnatal care interventions be adapted to effectively and cost-effectively support the specific health and nutritional needs of pregnant adolescents?	90.3	93.8	93.2	93.8	92.8	85.9
**2**	Description	What risk factors (biological, social, environmental etc.) impact the nutritional status of pregnant adolescents, including those in humanitarian contexts?	90.7	88.0	82.3	86.4	86.8	76.3
**3**	Delivery	What is the most acceptable and effective strategy in implementing daily multiple micronutrient supplementation for pregnant adolescents?	87.9	89.5	85.1	82.6	86.3	76.2
**4**	Description	What are the benefits of delaying early pregnancy on nutrition outcomes for adolescent girls?	89.8	84.2	80.6	83.8	84.6	75.4
**5**	Development	Are standard interventions that have been shown to be effective and cost-effective in all pregnant /postnatal women, equally effective and cost-effective in pregnant adolescents?	83.5	85.2	83.7	83.6	84.0	72.7

A=answerability, E=effectiveness, D=deliverability, Eq=equity. RPS=research priority score, AEA= average expert agreement. Ranking based on RPS.

### Other top rankings

As well as being the top ranked question overall, the question on adapting antenatal and postnatal care for pregnant adolescents was also ranked as having the greatest potential to improve equity (**Table 6**). The question related to strategies for effective delivery of interventions in schools was ranked the highest on answerability, as well as being most likely to result in an intervention that is effective, and deliverable. Since there were no research questions from the ‘discovery’ category in the top ten, the highest-ranking discovery question is also presented (**Table 6**).

**Table 6:** Top ranked questions according to priority-setting criteria.

CATEGORY	RESEARCH QUESTION
**MOST ANSWERABLE**	What strategies are effective for delivering interventions in schools to improve quality of diets and nutritional outcomes of SAC and adolescents?
**MOST EFFECTIVE**	What strategies are effective for delivering interventions in schools to improve quality of diets and nutritional outcomes of SAC and adolescents?
**MOST DELIVERABLE**	What strategies are effective for delivering interventions in schools to improve quality of diets and nutritional outcomes of SAC and adolescents?
**MOST EQUITABLE**	How should antenatal and postnatal care interventions be adapted to effectively and cost-effectively support the specific health and nutritional needs of pregnant adolescents?
**HIGHEST RANKED ’DISCOVERY’ QUESTION**	What standard indicators should be used to assess impact of nutrition interventions on school achievement, productivity, and wellbeing (e.g., mental, social, spiritual health) in SAC and adolescents?

There were small differences in the top ranked questions when results were disaggregated by respondent’s region of work (**S2 Table in S1 File**). Questions on delivery of in-school interventions ranked the highest in all regions with greater than 10 respondents (West and Central Africa, East and Southern Africa, and South Asia). However, there were some unique questions ranked top by region that are not represented in the overall top ten. For example, those working in South Asia ranked a development question exploring the effectiveness of integrating nutrition programmes with sexual and reproductive health interventions as the fifth highest priority. Those working in West and Central Africa ranked a delivery question on the cost-effectiveness of macronutrient supplementation for thin SAC and adolescents as the fourth highest priority question.

## Discussion

This CHNRI exercise brought together experts from diverse global regions and organisations to prioritise areas of research aiming to improve nutrition in SAC and adolescents in LMICs, over the next 10 years. Since many of the survey respondents were from programme-implementation backgrounds, these research priorities likely reflect practical issues being faced by these stakeholders. Overall, the top priority questions were similar across middle childhood, early adolescence, and late adolescence, and focused on development and delivery of interventions. This likely reflects the current knowledge base for these age groups, with efforts to date primarily focused on establishing their needs and advocating for their inclusion within global research, policy and programming agendas. As such, our findings indicate that future work should prioritise identifying and delivering effective interventions to reduce the burden of malnutrition, starting in schools.

A previous CHNRI exercise on adolescent health in LMICs conducted in 2016 had a section on nutrition [15]. Similarities exist between the research priorities published in 2016 and those identified in this CHNRI exercise, particularly relating to identifying delivery platforms, the benefits of delaying pregnancy in adolescent girls, and the development or tailoring of antenatal interventions to support the needs of pregnant adolescents. There are also some differences in the levels of priority given to determining the causes of anaemia in adolescent girls (highest ranked question in the 2016 CHNRI exercise) and describing the burden or prevalence of under- and overnutrition, which did not rank highly in our survey. This suggests that progress has been made in describing and understanding the burden of malnutrition and the causes of anaemia in adolescents since 2016. This is evidenced by the 2017 AA-HA! Report that cited iron deficiency anaemia as the leading cause of lost disability adjusted life years (DALYs) in adolescent girls 10-19 years and boys 10-14 years globally [16] and further supports the need to prioritise delivery and tailoring of effective interventions for SAC and adolescents.

The highest RPS in this survey was awarded to a question on adapting existing antenatal and postnatal services for pregnant adolescents. This is not surprising given that complications from pregnancy and childbirth are the leading cause of death for girls in this age group [17]. In many LMICs, early marriage for adolescent girls and/or adolescent pregnancy are major consequences of gender-based norms and discrimination, resulting in approximately 12 million girls 15-19 years giving birth each year [17, 18]. There is a general lack of specific guidance and tailored services despite these very high numbers, which also supports the need for increased access to suitable antenatal and postnatal care services for adolescent girls who become pregnant, as well as universal access to interventions aimed at reducing the risk of a first, or subsequent, early pregnancy [19]. This research question was also ranked highest on the potential to improve equity; adolescent marriage and pregnancy may perpetuate gender inequalities in educational attainment and economic opportunities and increase the risk of gender-based violence [20].

Another top ranked question focused on improvements that can be made to local food systems, with respondents ranking this question particularly high on effectiveness for prevention and management of malnutrition. Food systems are fundamental drivers of people’s diets, and the negative aspects of modern food systems disproportionately affect SAC and adolescents [21, 22]. Increasing attention has also been placed on transforming food systems in recent years, including at the 2021 United Nations Food Systems Summit that was underway around the time that respondents completed the CHNRI survey and may therefore have influenced the high prioritisation of this question. Food system transformations require a multisectoral approach, as do effective adolescent nutrition interventions [11, 21], which was recognised by the high ranking given to the question on ‘integrated packages of interventions’.

Strategies for delivering school-based interventions achieved high scores for answerability, deliverability, and effectiveness. Schools are regarded as having great potential as effective delivery platforms. Approximately 80% of SAC and adolescents globally are in school (UNESCO, 2019) and a recent literature review found that 95% of interventions targeting malnutrition in SAC and adolescents across LMICs were implemented in schools [10]. Similarly, research related to optimising school meals was ranked comparatively higher for younger children and adolescents who may have less active roles in making food choices and be more likely to attend school and receive school meals. While supporting improved nutrition outcomes for children and adolescents, school meals also promote enrolment and attendance at school in LMICs [23, 24]. Iron supplementation in schools may also have positive impacts on learning [25]. The importance of school meals was recently recognised during the 2021 Nutrition for Growth (N4G) summit, where school feeding, as well as anaemia prevention and treatment, were the most common commitments made by countries related to the 10-19 age bracket [26].

When looking at out-of-school children and adolescents, high ranked questions had comparatively lower deliverability scores, as well as some of the lowest AEA scores. Out-of-school children and adolescents constituted approximately 258 million of those 6-17 years of age in 2017 – a figure that has been exacerbated through the COVID-19 pandemic as a result of school closures [27, 28]. Out-of-school adolescents are harder to reach, often being a vulnerable and heterogeneous group, including married and pregnant adolescent girls and new mothers, as well as those in formal and informal employment, and may require innovative approaches for inclusive engagement [29]. Data from adolescent sub-groups such as those out-of-school, as well as refugees, those in humanitarian contexts, and boys in general [14] are lacking.

### Limitations

Many people who engaged with the survey completed their demographic information but did not answer any research priority questions. This may have been due to the length of the survey, that was made longer due to further disaggregating questions by age groups. While this disaggregation may have resulted in some survey fatigue, it was highly beneficial in allowing for comparison and highlighting similarities across age groups and identifying unique needs. Similarly, disaggregation by school status and inclusion of specific questions related to adolescent pregnancy will ensure that vulnerable, and sometimes neglected, groups can be prioritised in research agendas in the future. In comparing the demographic information of those who did and did not complete at least one section of the survey, few differences were found. The lack of description and discovery questions in the top 10 may have been influenced by the CHNRI methodology which is tailored towards prioritising research questions that are answerable and provide deliverable and effective results within a certain time period. Consequently, new, and more innovative research is unlikely to rank highly. Important research gaps which focus on improving infant health and nutrition through preconception and pregnancy interventions for adolescents were beyond the scope of this exercise which focused only on better nutrition outcomes for school-aged children and adolescents themselves. Lastly, the ‘4Ds’ framework, while adding standardised structure to the rankings, are subject to differences in interpretations, meaning that the 4D classification used in this study may differ from other CHNRI studies.

## Next steps

Taking these research priorities forward, implementation research for the top ranked delivery questions is needed from programme implementors, while engagement with academic institutions is needed for research to improve existing interventions. All research efforts need to include children and adolescents in the design, implementation, interpretation and application of results to ensure their appropriateness and increase the likelihood of success [30]. But this requires both consultation and meaningful youth engagement on how best to be inclusive. For age-disaggregated questions, the top five research questions were identical across the three age categories, which has practical benefits for research in reducing both the number of priority questions and the complexity of the overall research portfolio. While the questions may be the same, or similar, this does not necessitate similar approaches or answers across regions, age groups, or between in-school vs. out-of-school children and adolescents. It is likely that age-, pubertal stage- and context-specific research approaches and solutions are needed. Thus, it is important that future research takes place across a diverse set of regions, contexts, and that findings are disaggregated by sub-categories to identify nuances in the results.

## Supporting information

S1 Table Ranking results for all questions in the CHNRI exercise. (XLSX)

S1 File It contains all the other supporting tables. (DOCX)
