# Supplementary material for: Research priorities for nutrition of school-aged children and adolescents in low- and middle-income countries
Source: PLoS One. 2023 Jan 20;18(1):e0280510. doi: 10.1371/journal.pone.0280510 (PMC9858330; doi:10.1371/journal.pone.0280510)
Supplement: S1 File — (DOCX) [file pone.0280510.s002.docx]

**Supplementary Material for “Research priorities for nutrition of school-aged children and adolescents in low- and middle-income countries”**

**S1 Table: All questions ranked (see spreadsheet)**

**S2 Table: Regional results**

| **Region** | **Rank** | **Question number** | **QUESTION** | **N^o^ respondents** | **MEAN RPS** |
| --- | --- | --- | --- | --- | --- |
| West Africa | 1 | 17 | What are the optimal delivery platforms for reaching the sub-groups of SAC and adolescents identified as highest priority? | 15 | 95.8 |
|  | 2 | 18 | What are the optimal delivery platforms (health, education, social protection, media/technology etc.) for effective uptake of nutrition interventions for SAC and adolescents, taking into account scale, sustainability and youth engagement? | 12 | 95.8 |
|  | 3 | 19 | What strategies are effective for delivering interventions in schools to improve quality of diets and nutritional outcomes of SAC and adolescents? | 14 | 95.8 |
|  | 4 | 21 | What is the cost-effectiveness of macronutrient supplementation to thin SAC and adolescent girls and boys? | 14 | 95.8 |
|  | 5 | 42 | What risk factors (biological, social, environmental etc.) impact the nutritional status of pregnant adolescents, including those in humanitarian contexts? | 15 | 95.0 |
| East and Southern Africa | 1 | 19 | What strategies are effective for delivering interventions in schools to improve quality of diets and nutritional outcomes of SAC and adolescents? | 21 | 95.4 |
|  | 2 | 23 | What improvements can be made to local food systems to support access to healthy diets in schools? | 22 | 94.9 |
|  | 3 | 24 | What is the impact of peer education programmes on nutrition of adolescents and SAC in different contexts? | 22 | 94.9 |
|  | 4 | 18 | What are the optimal delivery platforms (health, education, social protection, media/technology etc.) for effective uptake of nutrition interventions for SAC and adolescents, taking into account scale, sustainability and youth engagement? | 21 | 94.4 |
|  | 5 | 47 | How should antenatal and postnatal care interventions be adapted to effectively and cost-effectively support the specific health and nutritional needs of pregnant adolescents? | 22 | 94.2 |
| South Asia | 1 | 19 | What strategies are effective for delivering interventions in schools to improve quality of diets and nutritional outcomes of SAC and adolescents? | 14 | 96.8 |
|  | 2 | 15 | What are effective, context-specific, behaviour change communication strategies to improve diets and nutritional status of SAC and adolescents? | 14 | 94.9 |
|  | 3 | 14 | What strategies are effective at involving SAC and adolescents in defining their own context-specific solutions to nutrition problems, and does their involvement result in more effective interventions? | 14 | 94.3 |
|  | 4 | 21 | What is the cost-effectiveness of macronutrient supplementation to thin SAC and adolescent girls and boys? | 14 | 93.9 |
|  | 5 | 32 | What is the effectiveness of integrating nutrition programs with sexual and reproductive health interventions (such as those aimed at delaying pregnancies or preventing HIV) on nutrition outcomes? | 13 | 93.3 |

Key: Green shading = specifically for in-school adolescents. Peach shading= specifically for pregnant adolescent girls. No shading= SAC and adolescents generally
